# A biosynthetic aspartate N-hydroxylase performs successive oxidations by holding intermediates at a site away from the catalytic center

**DOI:** 10.1016/j.jbc.2023.104904

**Published:** 2023-06-10

**Authors:** Laura Rotilio, Alessandro Boverio, Quoc-Thai Nguyen, Barbara Mannucci, Marco W. Fraaije, Andrea Mattevi

**Affiliations:** 1 Department of Biology and Biotechnology, University of Pavia, Pavia, Italy; 2 Molecular Enzymology, Groningen Biomolecular Sciences and Biotechnology Institute, University of Groningen, Groningen, The Netherlands; 3 Faculty of Pharmacy, University of Medicine and Pharmacy at Ho Chi Minh City, Ho Chi Minh City, Vietnam; 4 Centro Grandi Strumenti, University of Pavia, Pavia, Italy

**Keywords:** substrate recognition, monooxygenase, flavin, reaction mechanism, nitropropionate

## Abstract

Nitrosuccinate is a biosynthetic building block in many microbial pathways. The metabolite is produced by dedicated L-aspartate hydroxylases that use NADPH and molecular oxygen as co-substrates. Here, we investigate the mechanism underlying the unusual ability of these enzymes to perform successive rounds of oxidative modifications. The crystal structure of *Streptomyces* sp. *V2* L-aspartate N-hydroxylase outlines a characteristic helical domain wedged between two dinucleotide-binding domains. Together with NADPH and FAD, a cluster of conserved arginine residues forms the catalytic core at the domain interface. Aspartate is found to bind in an entry chamber that is close to but not in direct contact with the flavin. It is recognized by an extensive H-bond network that explains the enzyme’s strict substrate-selectivity. A mutant designed to create steric and electrostatic hindrance to substrate binding disables hydroxylation without perturbing the NADPH oxidase side-activity. Critically, the distance between the FAD and the substrate is far too long to afford N-hydroxylation by the C4a-hydroperoxyflavin intermediate whose formation is confirmed by our work. We conclude that the enzyme functions through a catch-and-release mechanism. L-aspartate slides into the catalytic center only when the hydroxylating apparatus is formed. It is then re-captured by the entry chamber where it waits for the next round of hydroxylation. By iterating these steps, the enzyme minimizes the leakage of incompletely oxygenated products and ensures that the reaction carries on until nitrosuccinate is formed. This unstable product can then be engaged by a successive biosynthetic enzyme or undergoes spontaneous decarboxylation to produce 3-nitropropionate, a mycotoxin.

Flavin-dependent monooxygenases form a large and ubiquitous class of enzymes with crucial roles in biosynthetic pathways and xenobiotic detoxification (1, 2). They are classified into six subclasses (A-F) based on their structural and mechanistic features (3, 4, 5). Members of the subclass B operate as single-component proteins, typically with excellent chemo-, regio-, and enantioselectivity properties (6). Their tightly bound flavin accepts reducing equivalents from nicotinamide cofactors, NADPH and/or NADH, and then reacts with molecular oxygen (Fig. 1*A*). This so-called “reductive activation” yields the unstable C4a-(hydro)peroxyflavin intermediate that inserts an oxygen atom into the substrate, the crucial step of the reaction. Formation of the ternary complex between the C4a-(hydro)peroxyflavin, NADP^+^ and substrate prevents the collapse of the intermediate and the concomitant unproductive generation of hydrogen peroxide (the so-called uncoupling). Examples of enzymes belonging to subclass B include flavin-containing monooxygenases (FMOs) (7, 8), type I Baeyer-Villiger monooxygenases (9) and N-hydroxylating monooxygenases (NMOs) (10, 11, 12).

**Figure 1:**
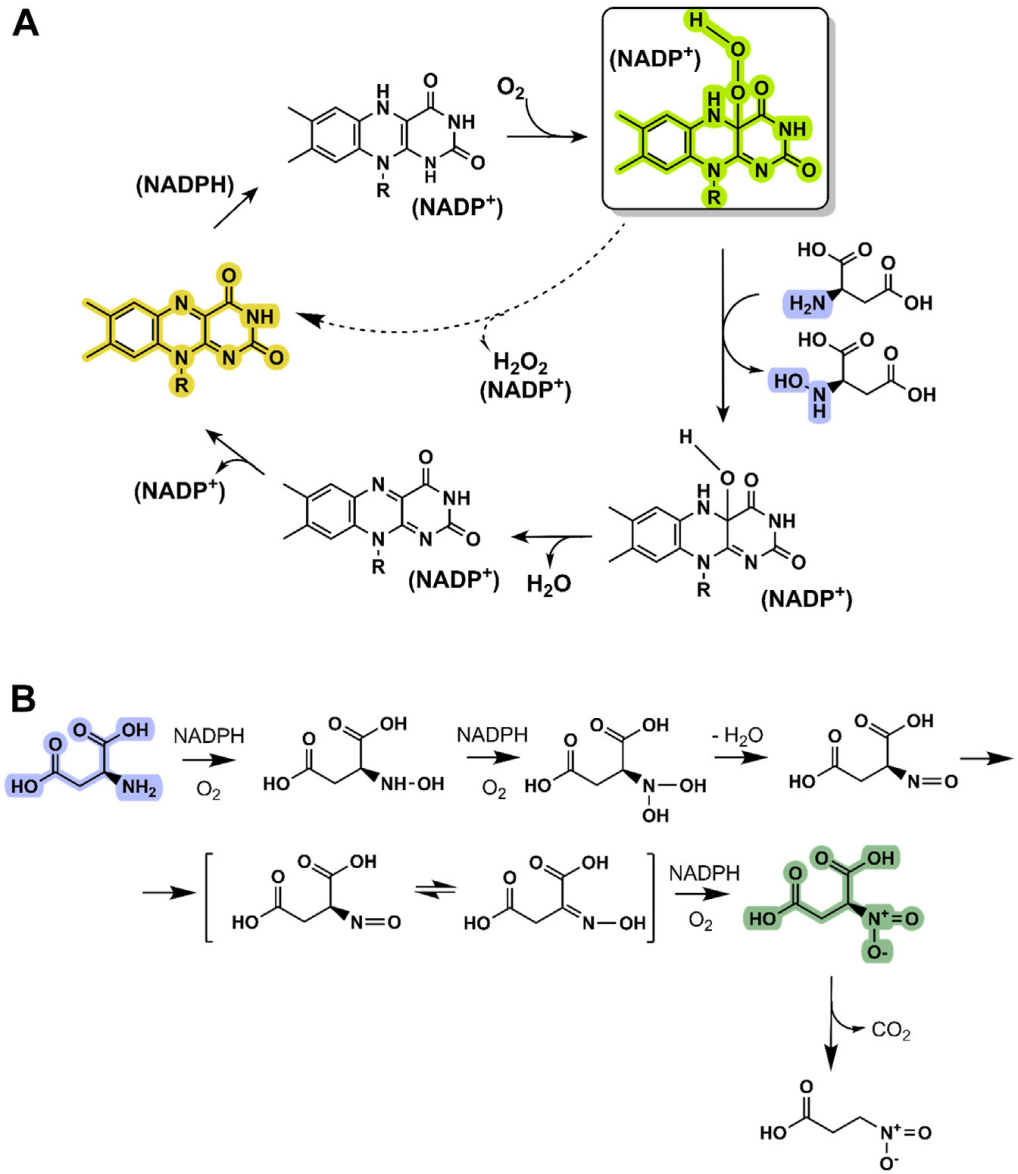
**Schematic representation of the overall catalytic cycle of flavin-dependent monooxygenases.***A*, the reaction starts with the reduction of the oxidized flavin (*yellow*) mediated by NAD(P)H. NA(D)P^+^ binding is essential for the stabilization of the C4a-hydroperoxyflavin intermediate (*green*) and to minimize the wasteful consumption of NADPH (*dashed line*). *B*, the reactions catalyzed by FzmM and similar L-aspartate hydroxylases. Conversion of L-aspartate to nitrosuccinate requires three NADPH molecules, suggesting a stoichiometry of 1:3 product:NADPH. The process may involve the spontaneous dehydration of the dihydroxy-aspartate to generate an oxime intermediate that can undergo the final third round of hydroxylation. Nitrosuccinate can spontaneously decarboxylate giving rise to 3-nitroproprionate (28).

The iconic members of the NMO family are PvdA and SidA, which were identified in *Pseudomonas aeruginosa* and *Aspergillus fumigatus*, respectively (13, 14). Despite being isolated from distant organisms, they both perform the same reaction: the conversion of L-ornithine to N5-hydroxy ornithine, the building block for the biosynthesis of hydroxamate-containing siderophores. A comprehensive structural analysis highlighted the distinctive features of this enzyme family whose structure shows the canonical double dinucleotide-binding domain architecture (15, 16, 17, 18). Characteristically, PvdA and SidA feature a small subdomain located in the proximity of the flavin and dedicated to the specific binding of L-ornithine. Such a strict substrate specificity sets the NMOs apart from the other class B monooxygenases that are typically endowed with a broader substrate profile (11).

These initial findings have been followed by an increasing interest in NMOs and many enzymes belonging to this family have been discovered in the past years (19, 20). A subgroup of NMOs that are attracting considerable interest are PcXL, FzmM, and its homolog CreE (21, 22, 23, 24, 25, 26). They were isolated from *Streptomyces* species and reported to participate in the biosynthesis of phosphonocystoximate, fosfazinomycin, and cremeomycin, respectively. Besides their largely different chemical structures, the biosynthesis of these natural products involves the di- or tri-hydroxylation of a nitrogen-containing precursor (Fig. 1*B*). Specifically, PcxL is an oxime-forming N-oxygenase that produces 2-hydroxyiminoethylphosphonic acid, the first step in the biosynthesis of oxime-containing natural products that are endowed with valuable biological activities (27). FzmM and CreE catalyze the triple N-hydroxylation of L-aspartate to nitrosuccinate. This unstable compound (28) is then utilized by a subsequent lyase that generates the nitrous acid (HNO_2_) employed in the biosynthesis of fosfazinomycin and cremeomycin (22). Similar hydroxylase/lyase enzyme pairs have been found in several microorganisms where they act as a HNO_2_-producing systems for the formation of N-N bonds containing natural products (29, 30).

Mechanistically, FzmM and PcxL are peculiar in that they catalyze multiple cycles of N-hydroxylation on the same substrate. This feature contrasts with the activities of the N-hydroxylating enzymes of the NMO and FMO families that generally catalyze only a single round of hydroxylation. Intrigued by this property, here we present the structural and mechanistic investigation of the L-aspartate N-hydroxylase from *Streptomyces* sp. *V2*. We propose a mechanism whereby the same substrate, L-aspartate, can be sequentially hydroxylated through its sequestration in an entry site that is adjacent to the flavin.

## Results and discussion

### Identification and purification of a suitable aspartate N-hydroxylase

The availability of stable enzymes was a prerequisite for our intended structural and mechanistic studies on the NMOs capable of multiple hydroxylations. Using as reference the sequences of PcxL and FzmM from *Streptomyces* strains, we identified eight enzyme candidates. They were selected to represent a sufficiently diverse enzyme panel, as predicted from the sequence identities and conservation of sequence motifs (Table 1) (22, 27). Each candidate was expressed in *Escherichia coli*, purified, and tested for activity on aspartate (FzmM substrate) or 2-aminoethylphosphonate (PcxL substrate) by assessing the rates of NADPH consumption (Fig. 1*A*). The enzyme from *Streptomyces* sp. *V2* stood out for its good expression levels and sustained activity, while all the other candidates were, at best, only moderately active with a tendency to lose activity over time. The protein is 54% identical in sequence to the previously characterized FzmM from *Streptomyces* sp. *XY332* (Table 1). The recombinant enzyme was purified in yellow fractions, indicative of flavin cofactor binding, and purified to high levels as evaluated by SDS-PAGE (Fig. S1). The protein yields were about 15 mg protein/L cell culture. The native molecular weight was evaluated using size exclusion chromatography and mass photometry and estimated to be 65 kDa after cleavage of the His_8_-SUMO purification tag. The UV-visible absorbance spectrum showed two characteristic peaks at 370 and 456 nm, further indicating that the oxidized cofactor was bound in the active site (Fig. S1). The enzyme C-terminal residues were predicted to be disordered by AlphaFold (31) (Fig. S2). Therefore, we also considered a C-terminally truncated mutant lacking the amino acids 602 to 616. This variant featured the same expression levels and activity properties of the wild-type enzyme (Table 2). It was employed for further biochemical and structural studies, considering that it readily crystallized, unlike the wild-type protein.

**Table 1:** *N*-hydroxylating monooxygenase candidates

Source of origin	Accession code^a^	Sequence identity with FzmM^b^ (%)	Sequence identity with PcxL^c^ (%)
*Streptomyces* sp. *V2*	PWG10292.1	54	17
*Streptomyces* sp. *NRRL WC-3744*	WP_030990682.1	17	58
*Streptomyces cremeus*	ALA99202.1	50	22
*Streptomyces roseus*	WP_048477421.1	48	21
*Kitasatospora azatica*	WP_035850953.1	47	17
*Streptomyces griseoruber*	KUN87143.1	52	19
*Streptomyces janthinus*	WP_193477728.1	53	19
*Streptomyces* sp. *NBRC 110468*	WP_078890443.1	52	18

a Database at the National Center for Biotechnology Information.

b WP_053787792.1.

c WP_051704824.1.

**Table 2:** Steady state kinetic parameters of *Streptomyces spV2* L-aspartate N-hydroxylase^c^

	Varying substrate	Constant substrate^a^	*k*_cat_ (s^−1^)	*K*_M_ (μM)
NADPH consumption				
Full-length	NADPH	−	0.19 ± 0.004	5.18 ± 0.82
	L-aspartate	NADPH	2.80 ± 0.26	288 ± 33
Wild type^b^	NADPH	−	0.13 ± 0.003	4.63 ± 0.47
	NADPH	L-aspartate	6.22 ± 0.34	30.47 ± 5.4
	L-aspartate	NADPH	7.05 ± 0.18	274 ± 27
N413A^b^	NADPH	−	0.15 ± 0.003	2.70 ± 0.33
	L-aspartate	NADPH	0.83 ± 0.048	>1000
V571F^b^	NADPH	−	0.18 ± 0.014	3.9 ± 1.16
	L-aspartate	NADPH	5.74 ± 0.184	>4000
Oxygen consumption				
Wild type	NADPH	L-aspartate	7.56 ± 0.28	23.05 ± 3.48
	L-aspartate	NADPH	7.17 ± 0.22	484 ± 67

a 100 μM NADPH or 1 mM L-aspartate.

b C-terminally truncated (Δ602–616) protein.

c Mean and standard deviation from three measurements.

### Kinetics analysis outlines a classical class B monooxygenase mechanism

**Figure 2:**
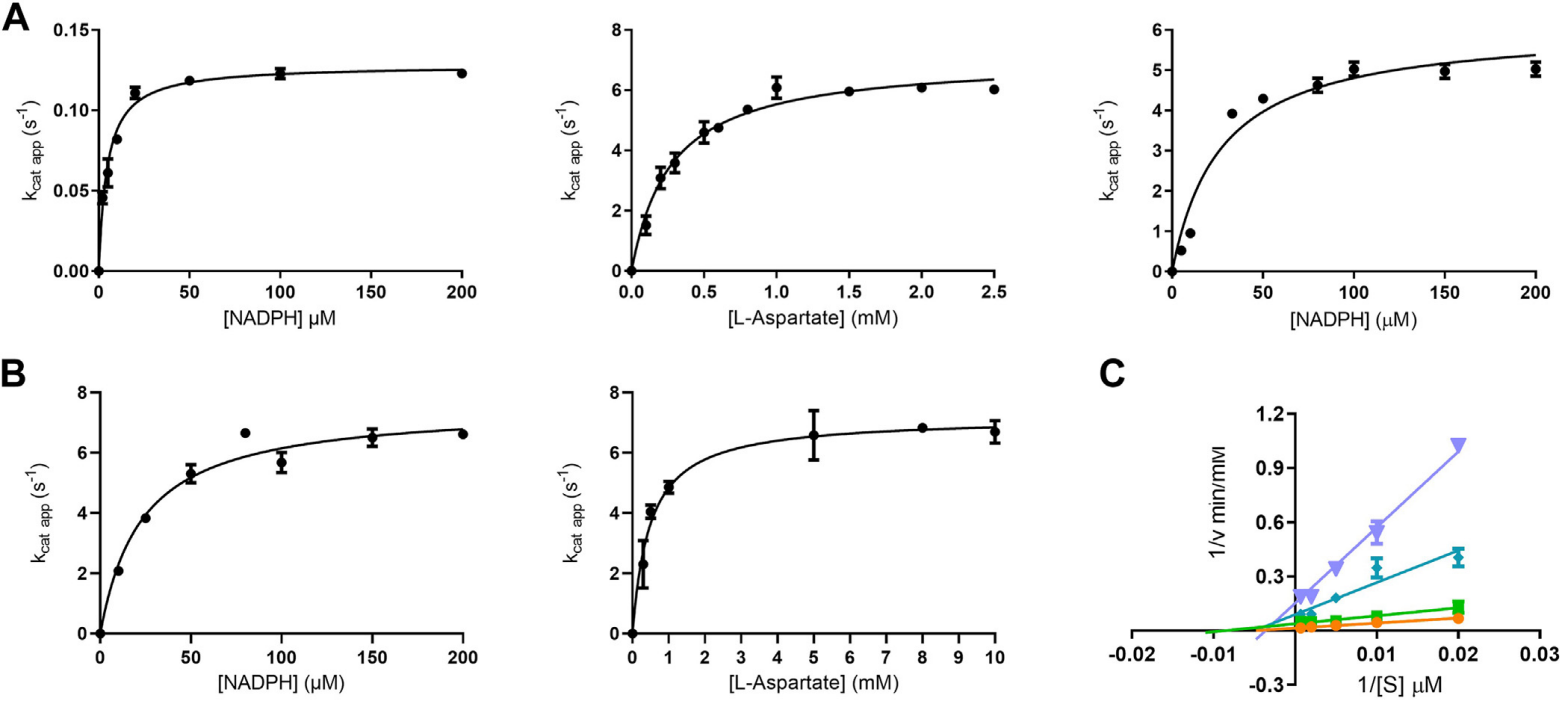
**Steady-state kinetics of *Streptomyces sp. V2* L-aspartate N-hydroxylase.** Michaelis-Menten kinetics by measuring (*A*) NADPH or (*B*) oxygen consumption. *C*, lineweaver-Burk plot. The NADPH concentrations are 5 μM (*orange*) - 2 μM (*green*) - 1 μM (*cyan*) – 0.5 μM (*purple*) whereas the L-aspartate concentrations ([S]) are 0.1 to 0.2 to 0.5 to 1.5 μM.

The steady-state kinetic parameters for the *Streptomyces* sp. *V2* aspartate N-hydroxylase were determined using the NADPH consumption assay (Fig. 2*A*). The NADPH oxidase activity (the so-called uncoupling) showed a *k*_cat_ of 0.13 s^−1^. The preference for the reducing cofactor was also evaluated and no turnover was detected with NADH. In the presence of L-aspartate, the enzyme showed a 60-fold increase in activity with a *k*_cat_ of about 7 s^−1^ and *K*_M_ values of 30 μM and 274 μM for NADPH and L-aspartate, respectively. By contrast, when testing L-glutamate, L-serine, L-threonine, D-aspartate, and 2-aminoethyl phosphonic acid (PcxL substrate), no activity boost was detected as the *k*_cat_ values observed for these compounds were equivalent to the rate of uncoupling. Very similar results were obtained by probing the oxygen consumption (Fig. 2*B* and Table 2). The Lineweaver-Burk plot of the initial rates revealed a pattern of lines intercepting on the horizontal axis, suggestive of a ternary complex mechanism. Thus, *Streptomyces* sp. *V2* L-aspartate N-hydroxylase operates as a typical class B flavoenzyme monooxygenase: it is a single-component enzyme that binds simultaneously FAD and NADPH and retains the pyridine nucleotide coenzyme throughout the catalytic process (Figs. 1*A* and 2*C*) (5).

**Figure 3:**
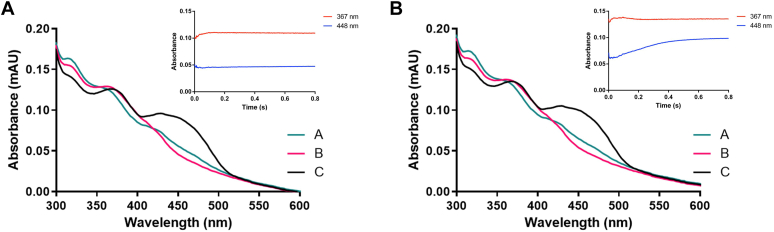
**Oxidative half-reaction of *Streptomyces sp. V2* L-aspartate N-hydroxylase**. NADPH-reduced enzyme (12 μM) was reacted with oxygen (130 μM) in (*A*) the absence and (*B*) the presence of L-aspartate (1 mM). All reactions were performed in 50 mM Hepes pH 8, 50 mM NaCl. The insets show the stopped-flow traces at 367 (*red*) and 448 (*blue*) nm.

To interrogate the intermediates behind oxygen activation and L-aspartate hydroxylation, stopped-flow measurements were performed using a photodiode array detector. After anaerobic incubation with 1 to 1.5 equivalents of NADPH, the reduced enzyme was mixed with an aerobic buffer in the absence and presence of L-aspartate. As shown in Figure 3, the deconvoluted spectra were fitted into a two steps A → B →C process. In the absence of the substrate, the first step occurs quickly (k_1_= 41.7 ± 0.3 s^−1^) leading to the C4a-hyroperoxyflavin with a maximum absorbance at 360 to 390 nm. The second step is much slower and leads to partial re-oxidation of the enzyme (k_2_= 0.6 ± 0.1 s^−1^). In the presence of 1 mM L-aspartate, the re-oxidation process was still composed of two phases. The first one (k_1_=37.1 ± 0.3 s^−1^) maintained a similar rate compared to the value measured in the absence of the substrate whereas the second phase (k_2_=4.2 ± 0.1 s^−1^) was seven times faster. This observation documented how the presence of L-aspartate positively affects the reaction rate, triggering the decomposition of the hydroperoxyflavin.

Collectively, these experiments indicated that *Streptomyces* sp. *V2* enzyme has a narrow substrate specificity as it is active only on L-aspartate. Furthermore, it exhibits a low degree of uncoupling as the NADPH consumption in the absence of L-aspartate is comparatively low, reflecting the slow decay of the C4a-hydroperoxyflavin (Fig. 1*A*). Overall, these kinetics and spectral properties closely resemble those originally described for FzmM from *Streptomyces* sp. *XY332* (23).

### 3-Nitropropionate is the prevailing reaction product

**Figure 4:**
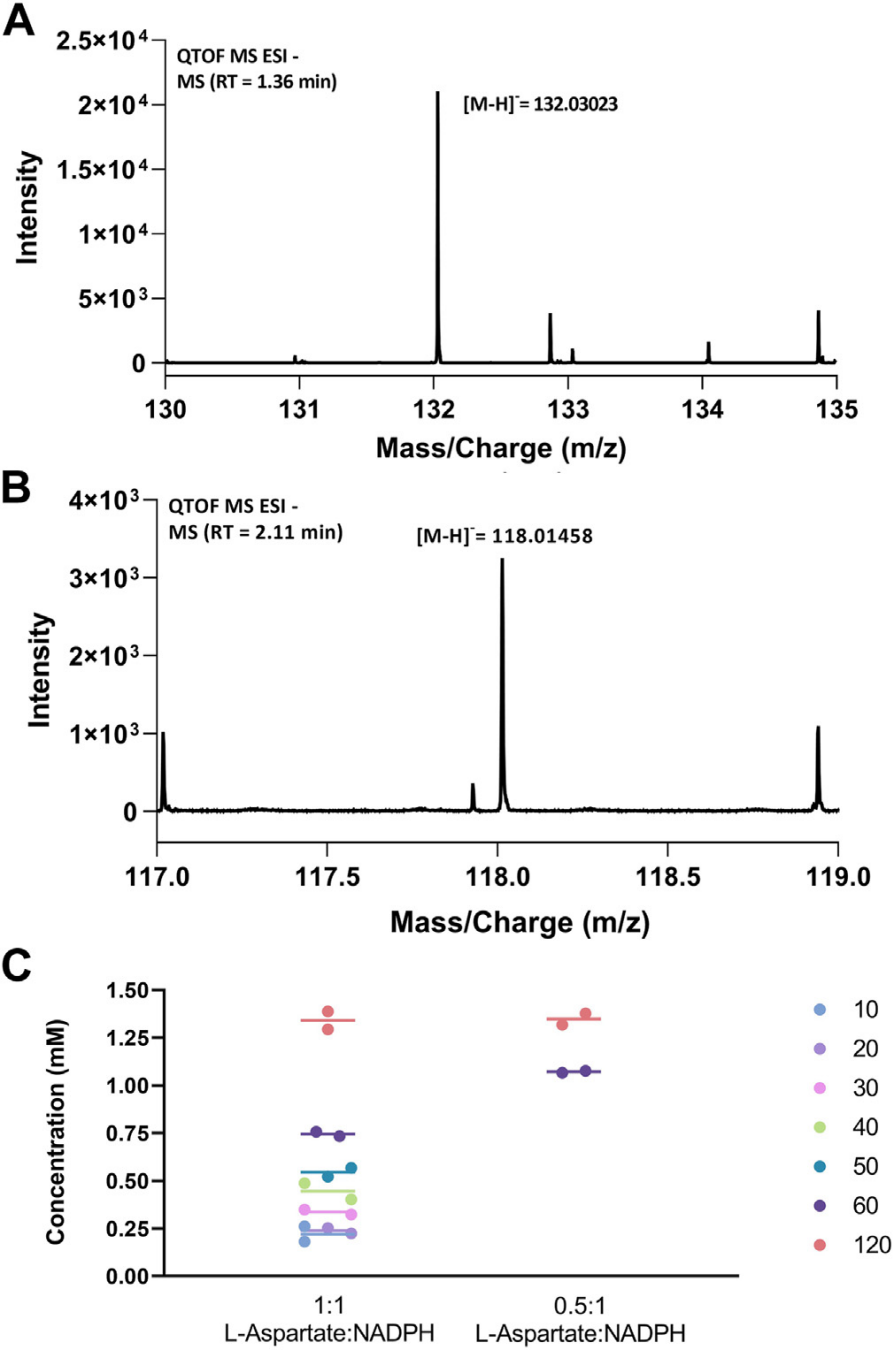
**UHPLC-HRMS (negative mode) analysis of *Streptomyces sp. V2* L-aspartate N-hydroxylase reaction mixture.** ESI-MS^-^ spectra of the [M-H]- ions for (*A*) L-aspartate (m/z = 132.03023; theoretical m/z = 132.03) and (*B*) 3-nitropropionate (m/z = 118.01458; theoretical m/z = 118.07). *C*, quantitative analysis of the 3-nitropropionate concentration at different time points (minutes). The samples contained 10 μM L-aspartate N-hydroxylase, 5 mM or 2.5 mM L-aspartate, and 5 mM NADPH in 50 mM Hepes pH 8. The reaction was carried out for 120 min at 25 °C with 180 rpm shaking. See Figs. S3–S5 for the complete analysis.

We analyzed the reaction products with ultra-high-pressure liquid chromatography coupled to high-resolution mass spectrometry. The reaction mixtures were inspected along with the analytical standards and controls without NADPH or the enzyme (Figs. S3 and S4). Critically, the sole and unique product found in the complete reaction mixture was identified as 3-nitropropionate having a m/z of 118.01458 Da (Figs. 4, *A* and *B* and S5). The reaction mix was exposed to fluorenylmethyloxycarbonyl chloride (Fmoc-Cl) and 1-adamantylamine to derivatize any labile hydroxyl-aspartate intermediate, but we could not identify any additional chemical species (22). We next monitored the accumulation of 3-nitropropionate with time using 1:1 and 0.5:1 L-aspartate:NADPH molar ratios. In both cases, the final 3-nitropropionate concentration was slightly less than one-third of the initial NADPH concentration (about 1.45 mM 3-nitropropionate from 5 mM NADPH and 5 mM or 2.5 mM L-aspartate; Fig. 4*C*). In summary, these data demonstrated that the most abundant reaction product is 3-nitropropionate which likely results from the spontaneous decarboxylation of the enzyme-generated nitrosuccinate (Fig. 1*B*). No mono- or di-hydroxylated intermediates appear to accumulate in the reaction mix. The process requires three rounds of hydroxylation and therefore occurs at the expense of three NADPH molecules. This agrees with the observed 3:1 ratio between the concentrations of the initial NADPH and final product, and the minimal levels of uncoupling.

### Crystal structure of the aspartate N-hydroxylase

**Figure 5:**
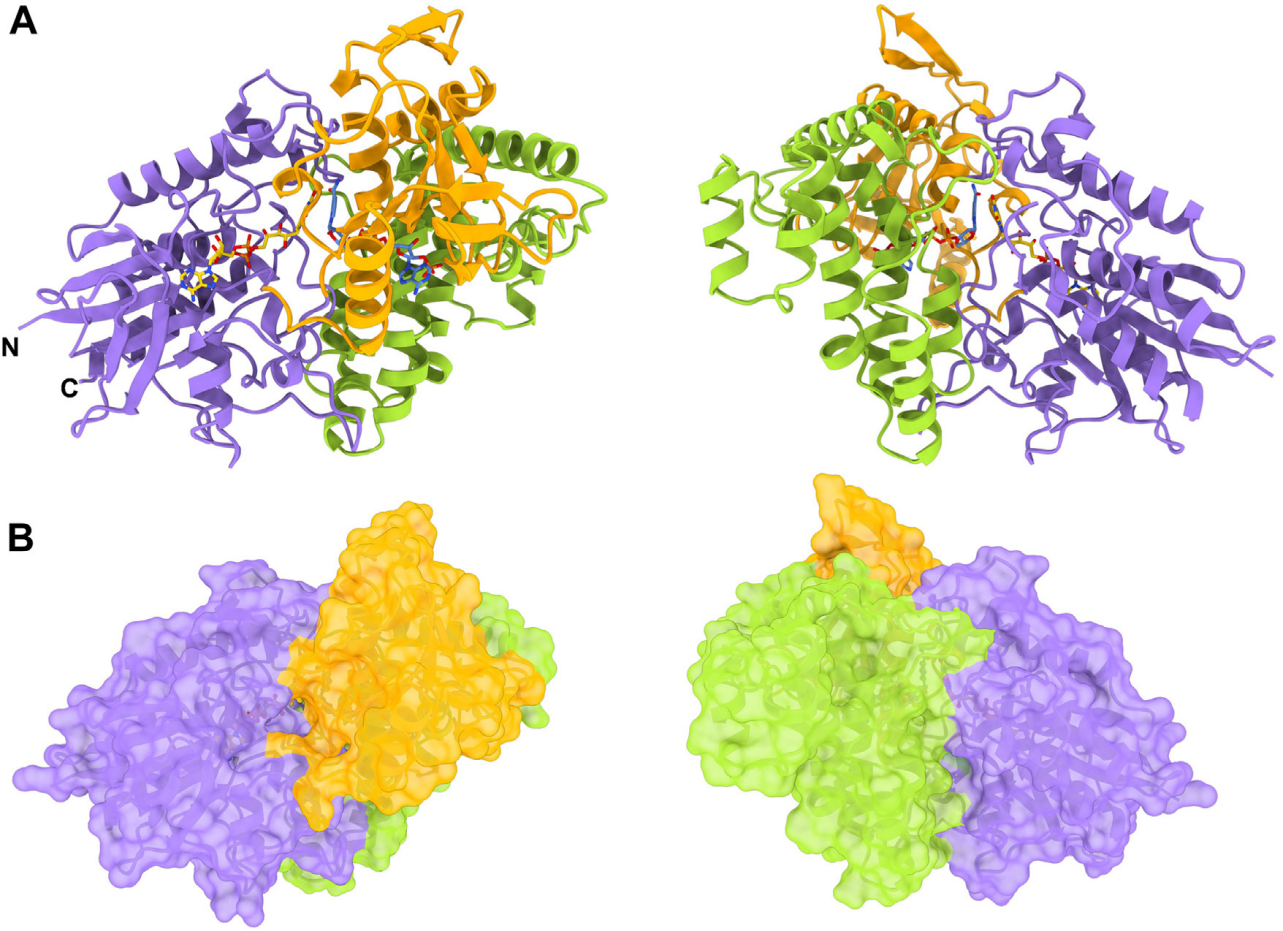
**Crystal structure of L-aspartate N-hydroxylase**. *A*, ribbon diagram and (*B*) molecular surface of the overall conformation. FAD- (residues 2–169/505–600), NADP- (170–263/448–594), and substrate-binding (264–447) domains are depicted in *purple, orange*, and *green*, respectively. FAD and NADP^+^ carbon atoms are in *gold* and *cornflower blue*. “N” and “C” outline the N- and C-termini.

**Figure 6:**
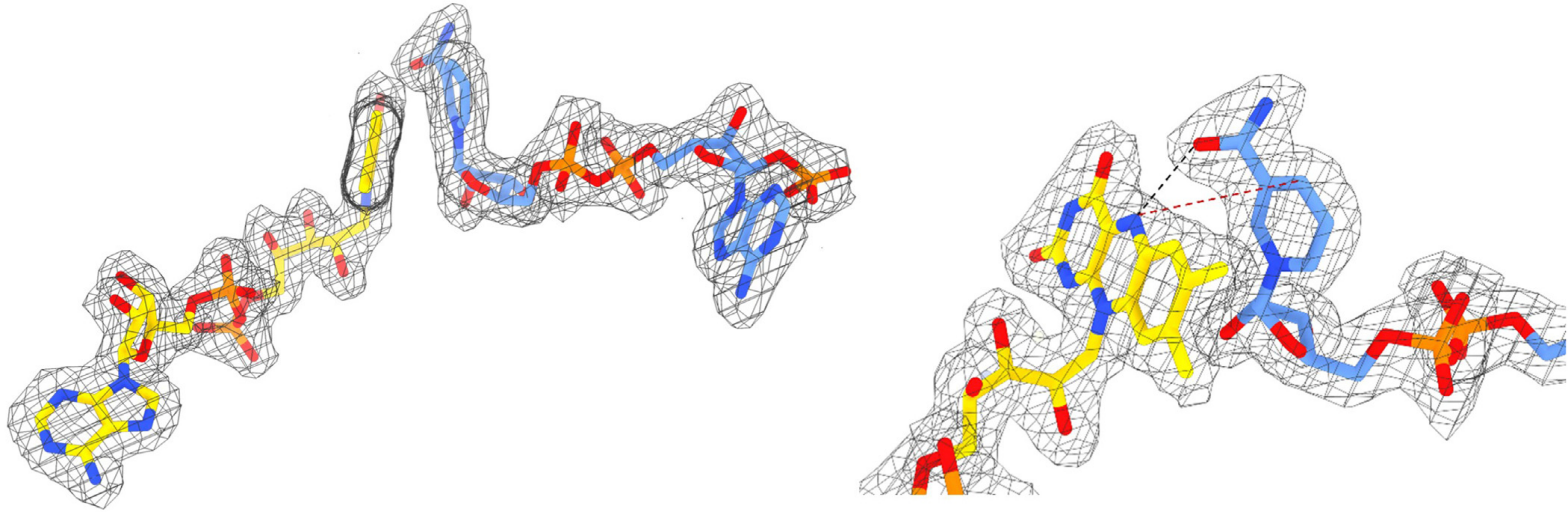
**Close-up view of the cofactors arrangement.** The H-bond between the nicotinamide carboxyl oxygen and the N5 of the reduced flavin is shown as a *black dashed line*. The *red dashed line* connects the reactive C4 and N5 atoms of the two cofactors. The weighted 2Fo-Fc electron density map is contoured at 1.5 σ level. The two views are rotated by about 90° around the *top-to-bottom* axis.

**Figure 7:**
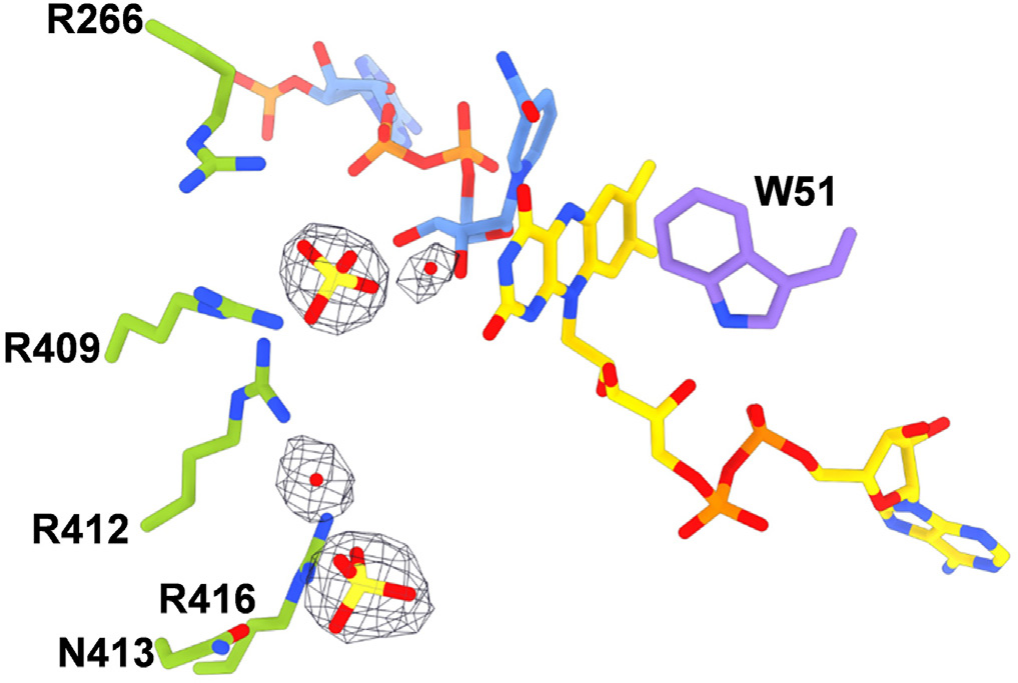
**Sulfate molecules bound to the active site.** Residues in *green* and *purple* belong to the substrate and FAD domains, respectively. The weighted 2Fo-Fc electron density is contoured at 1.5 σ level.

Knowledge of the three-dimensional structure became of interest to gain further insight into the substrate binding and reaction mechanism. The crystal structure of the enzyme with NADP^+^ was hence solved at 2.5 Å resolution (Table 3). The enzyme is a monomer comprising 22 α-helices and 17 β-strands arranged in a three-domain architecture (Fig. 5*A*) (16, 17, 32). The active site is in a cleft at the interface of the three domains (Fig. 5*B*) and both FAD and NADP^+^ are clearly defined by the electron density (Fig. 6). An intricate network of H-bonds interactions surrounds the two cofactors (Fig. S6). The isoalloxazine is sandwiched between the nicotinamide ring and Trp51 which provides a strong coordination through an edge-to-face interaction (Fig. 7). The nicotinamide ring positions its reactive C4 atom at 5.0 Å distance from the flavin N5 which is in contact with the nicotinamide carbamide group (Fig. 6). This arrangement is identical to the conformation observed in the structures of class B monooxygenases, such as PvdA, SidA, FMOs, and BVMOs (16, 33, 34).

**Table 3:** Crystallographic statistics

Aspartate-N-hydroxylase complex	Sulphate (8CP5)	L-Aspartate (8CP2)
Resolution range	60.5–2.5	53.4–2.1
Space group	H3	H3
Unit cell axes (Å,°)	243.68 243.68 126.30 90 90,120	244.59 244.59 128.11 90 90,120
Total reflections^a^	993,652 (40,976)	1,761,931 (183,162)
Unique reflections^a^	91,647 (8873)	166,775 (16,750)
Multiplicity^a^	10.8 (10.3)	10.6 (10.9)
Completeness^a^ (%)	99.36 (96.11)	99.96 (99.98)
Mean I/sigma(I)^a^	10.1 (0.6)	8.77 (1.39)
Wilson B-factor	55.62	29.31
R-merge^a^^b^	0.184 (0.91)	0.2504 (1.988)
CC_1/2_^a^^c^	0.996 (0.303)	0.994 (0.506)
Reflections used in refinement^a^	91,647 (8873)	166,753 (16,751)
R-work^a^	0.204 (0.3978)	0.179 (0.305)
R-free^a^	0.242 (0.3902)	0.202 (0.316)
N. of non-hydrogen atoms	9639	10,375
macromolecules	9286	9321
ligands	284	258
solvent	69	796
Protein residues	1199	1203
rmsd (bonds) (Å)	0.014	0.016
rmsd (angles) (°)	1.99	2.10
Ramachandran favored (%)	96.15	96.57
Ramachandran allowed (%)	3.85	3.43
Ramachandran outliers (%)	0.00	0.00
Average B-factor	53.40	36.23
macromolecules	53.04	35.97
ligands	68.74	37.88
solvent	39.47	38.79

a Values in parentheses are for the reflections in the highest resolution shell.

b R_merge_=∑|I_i_-<I>/∑I_i_, where I_i_ is the intensity of the i^th^ observation and <I> is the mean intensity of the reflection.

c The resolution cut-off was set to CC_1/2_ > 0.3 where CC_1/2_ is the Pearson correlation coefficients of the two “half” data sets, each derived by averaging half of the observations for a given reflection.

**Figure 8:**
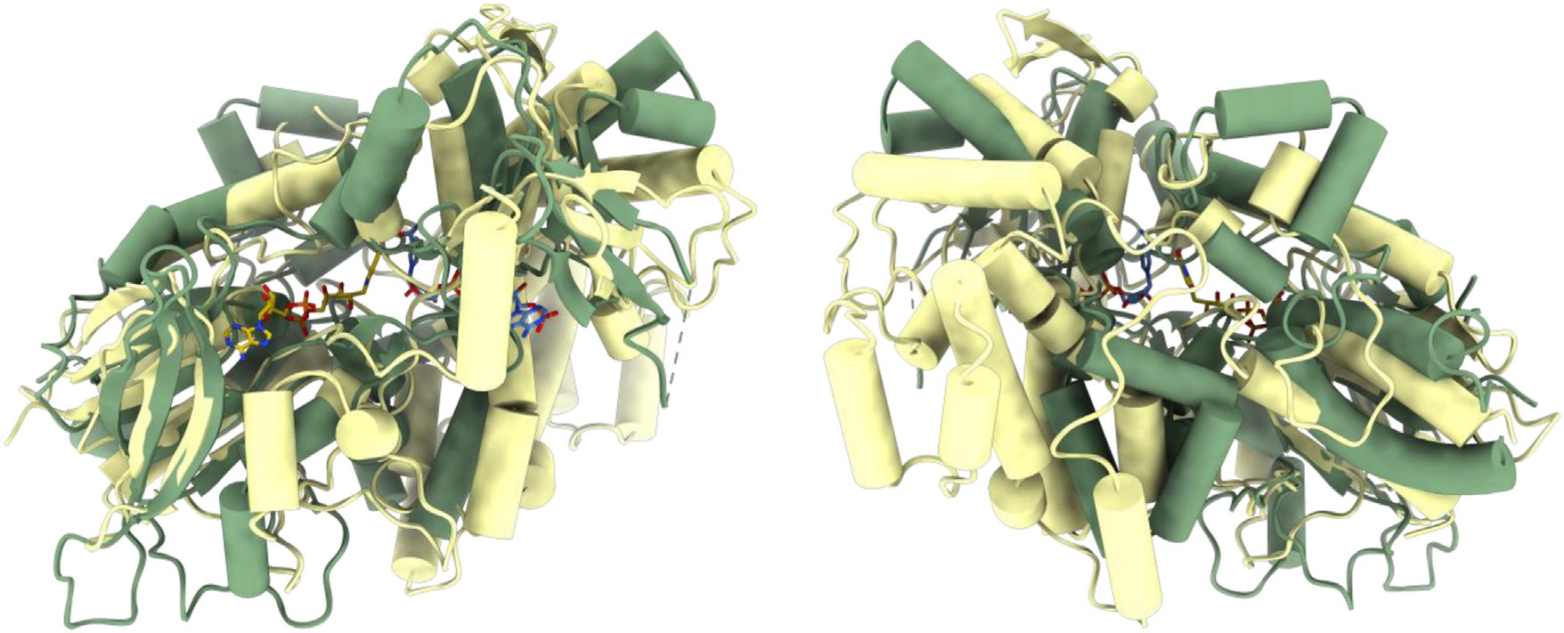
**Structural comparison with SidA**. Superposition between L-aspartate N-hydroxylase (*light yellow*) and SidA (*green*; PDB code 4B69) highlights major differences between the two structures. The two views are rotated by 180° around the *top-to-bottom* axis. The larger substrate-binding domain of L-aspartate hydroxylase is more clearly visible in the orientation of the *right panel*.

Consistent with the similarity in cofactor binding, a DALI search for structural homologs outlined SidA as the closest protein (17, 35). *Streptomyces* sp. *V2* L-aspartate N-hydroxylase and SidA share minimal (11.4%) sequence identity and superpose with an r.m.sd of 3.8 Å over 366 Cα atoms. The comparison shows that the two enzymes comprise very similar β-sheet cores in their FAD and NADP domains but differ for the lengths and orientation of the more external α-helices (Fig. 8). Moreover, the helical substrate-binding domain of the L-aspartate hydroxylase is bigger and can be hardly superposed onto the substrate domain of SidA. Therefore, the two enzymes are relatively distant in their three-dimensional structures besides sharing the quintessential FAD- and NADP-binding sites typical of the NMOs.

### An entry chamber for L-aspartate binding

The enzyme was originally crystallized in conditions where the sole precipitant in the reservoir solution was ammonium sulfate. Expectedly, the protein structure is populated with sulfate ions. Two of these sulfates were found juxtaposed to the FAD and embedded by an arginine-rich site, establishing H-bond interactions with Arg266, Arg409, Arg412, Asn413, and Arg416 (Fig. 7). Notably, these residues belong to the helical substrate-binding domain (Figs. 5*B* and 8). Being negatively charged molecules, we speculated that these ions occupy the putative position of the substrate and therefore inhibit the protein. Consistently, we found that the enzyme loses 50% of its activity already at 5 mM SO_4_^2-^ and becomes completely inhibited at 10 mM concentration. Interestingly, this effect is specific for sulfate because chloride (up to 100 mM) and phosphate (up to 50 mM) salts did not affect the enzyme. This finding highlighted the bound sulfate ions as likely substrate mimics and the associated Arg cluster as the putative site for L-aspartate binding.

**Figure 9:**
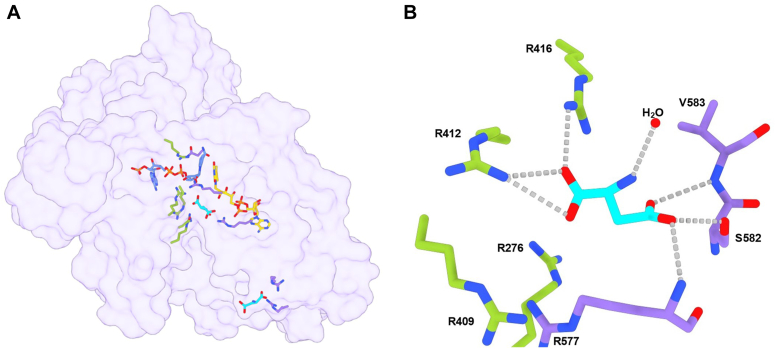
**Aspartate N-hydroxylase in complex with L-aspartate**. *A*, semi-transparent molecular surface showing the two L-aspartate molecules bound to the enzyme. FAD, NADP(H) and L-aspartate carbon atoms are in *gold*, *cornflower blue*, and *cyan* respectively. The peripheral L-aspartate interacts with Arg593 and Arg597. *B*, close-up view of L-aspartate binding in the entry chamber. Displacement of the water molecule bound to the substrate amino group would give room for binding an N-hydroxylated/oxygenated aspartate (Fig. 1*B*). Residues belonging to the FAD and substrate domains are in *purple* and *green*.

**Figure 10:**
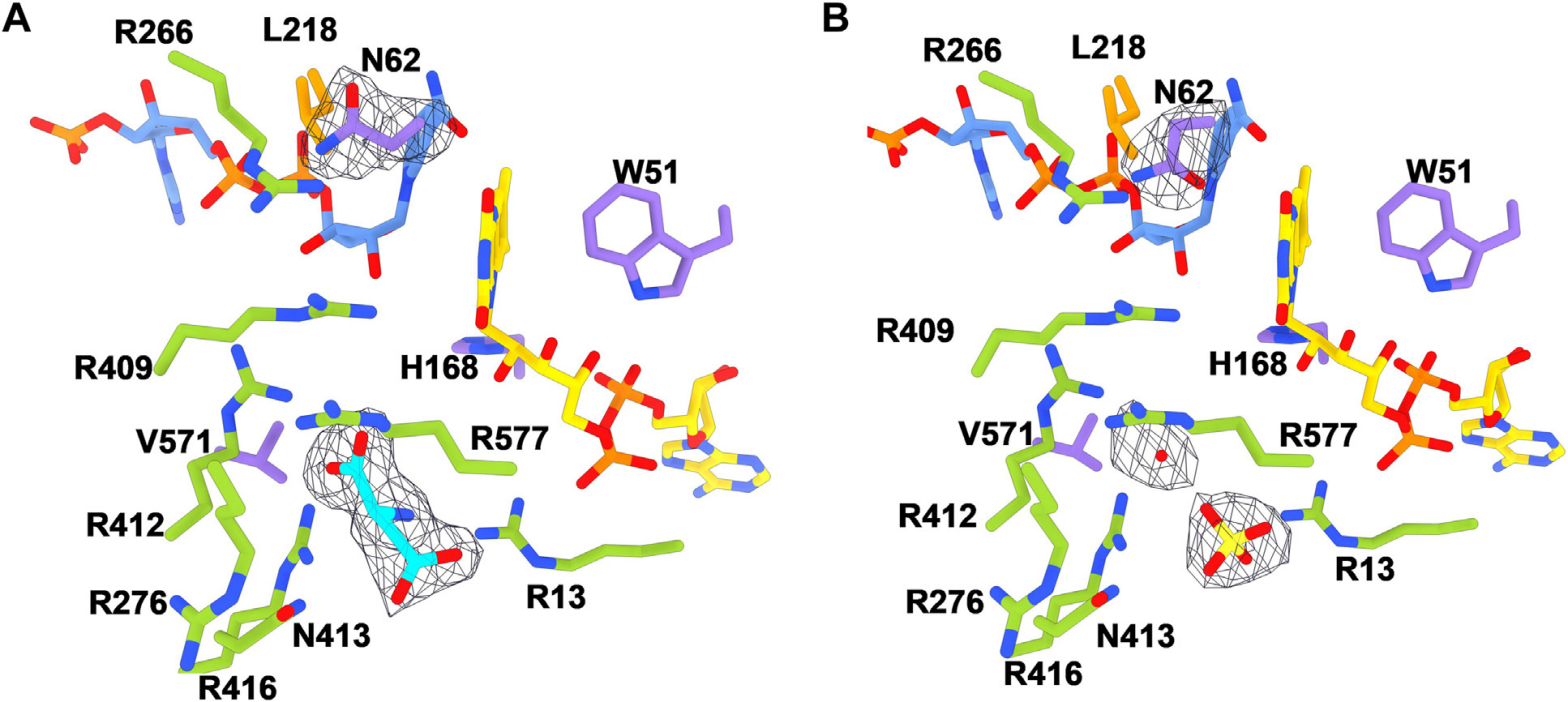
**Comparison between structures in complex with sulfate and L-aspartate**. The two structures are identical (r.m.s.d of 0.3 Å for all Cα atoms) except for the Asn62 side chain being (*A*) “inwards” and (*B*) “outwards” in absence and presence of the substrate, respectively. The weighted 2Fo – Fc electron density maps are contoured at 1.4 σ level. Residues belonging to FAD, NADP, and substrate domains are in *purple, orange*, and *green*.

The C-terminally truncated (see above) enzyme was extremely prone to crystallize in the presence of ammonium sulfate. Nonetheless, we noticed that the crystals could stand prolonged soakings in solutions comprising sodium tartrate, PEG4000, 15 mM L-aspartate, and 5 mM NADPH with no ammonium sulfate being present. We further observed bleaching of the crystals indicating that the enzyme was reduced by NADPH. This procedure thereby allowed us to solve the structure of the reduced enzyme in complex with NADP(H) and L-aspartate at 2.1 Å resolution. The electron density unambiguously showed the presence of a molecule of L-aspartate with the side chain carboxylate overlapping the position occupied by a sulfate ion (Figs. 9 and 10). The ligand is engaged by several interactions mainly with the above-described arginine cluster (Arg13, Arg276, Arg416, Arg577, and Asn413). The critical observation is that this position of L-aspartate cannot be conducive to the oxygenation of its amino group which remains too far away (∼10 Å) from the flavin N5-C4a locus. Therefore, we shall refer to this site as an “entry chamber”. For oxygenation to occur, the substrate should bind more deeply, inside the “catalytic” center wedged between the isoalloxazine and the nicotinamide-ribose moiety of NADP(H). We could not detect any bound L-aspartate in this site even after prolonged soaking. The catalytic center is instead occupied by a water molecule and further comprises Asn62 whose side chain lays on top of the flavin C4a. This residue is generally conserved or conservatively replaced by Asp or Glu among class B monooxygenases and demonstrated to be critical for catalysis in flavin-containing monooxygenases (FMOs) and Baeyer-Villiger monooxygenases (7, 36). We noticed a difference in the conformation of this side chain between the sulfate and L-aspartate bound structures. Its side chain is oriented “inwards” in the sulfate complex to partially occupy the catalytic site (Fig. 10*B*). In the L-aspartate structure, Asn62 is flipped “outwards”, vacating the space in front of the flavin (Fig. 10*A*). This small—yet significant—conformational change may have a catalytic role, as discussed below. Of interest, we also found a second L-aspartate bound to Arg593 and Arg597 at the entrance of the tunnel leading to the entry chamber. This ligand position probably identifies the passageway for substrate diffusion toward the catalytic core of the protein (Fig. 9*A*).

**Figure 11:**
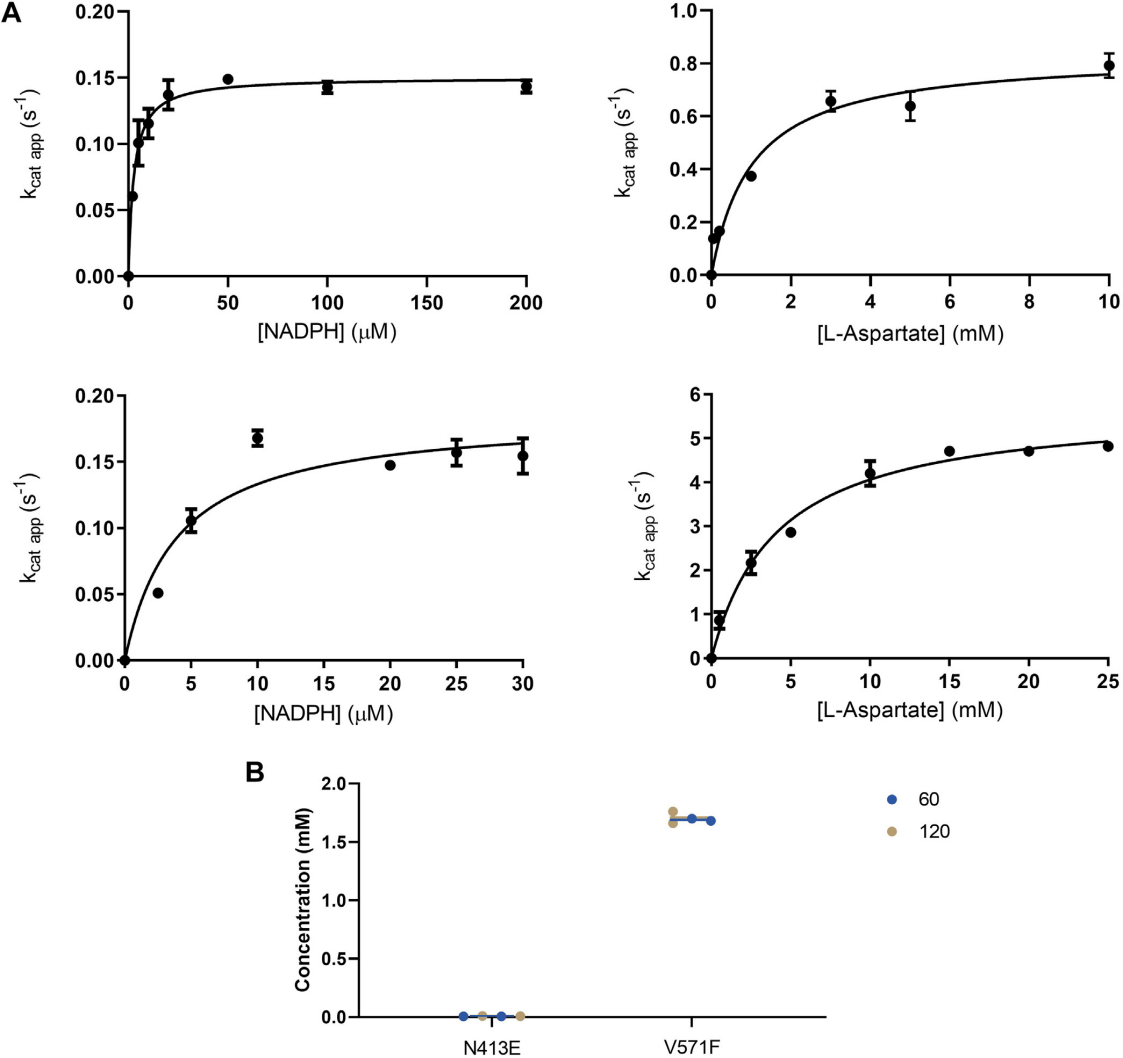
**Biochemical characterization of the N413E and V571F variants.***A*, michaelis-Menten kinetics of N413E (*top*) and V571F (*bottom*). *B*, 3-Nitropropionate quantification in the N413E and V571F reaction mixtures. All assays were carried out for wild-type enzymes (see Figs. 4*C* and S5).

The structural data raised the question of the functional role of the entry chamber in catalysis. To address this issue, we considered Asn413 which is located in the rear of the chamber, away from the catalytic site (Fig. 10*A*). We reasoned that mutating this residue to glutamate should probe the catalytic role of the entry chamber by drastically affecting L-aspartate binding through steric hindrance and electrostatic repulsion. The N413E mutant was expressed and purified as the wild-type enzyme. Its steady-state kinetic characterization indicated that the mutation hardly affected the NADPH oxidase activity as outlined by *k*_cat_ and *K*_M_ values almost identical to those exhibited by the wild-type enzyme (Fig. 11*A* and Table 2). Conversely, the mutant protein showed little L-aspartate hydroxylation activity. This was apparent from the limited 4-fold effect on the rate of NADPH consumption by the addition of L-aspartate. Moreover, no 3-nitropropionate or other hydroxylated products (*e.g.* L-hydroxyaspartate) were detected (Figs. 11*B* and S5). Thus, the N413E abolished hydroxylation while preserving NADPH oxidation, suggestive of a critical role in catalysis and/substrate binding by the entry chamber. To provide additional support to this notion, we considered Val571, located in the tunnel lined by NADPH and leading to the active site (Fig. 10). Val 571 was substituted with a bulkier and more hydrophobic phenylalanine with the intent of blocking the direct diffusion of L-aspartate to the catalytic site through a route that does not cross the entry chamber. The enzyme preserved the same level of NADPH oxidase activity as the wild-type protein (Fig. 11*A* and Table 3). Moreover, it remained capable of L-aspartate hydroxylation though with an increased *K*_M_ of 4 mM for the substrate (Fig. 11*B*, Table 3 and Fig. S5). Hence, the V571F mutation had limited effects contrary to the drastic impact caused by targeting the entry site. These mutagenesis experiments demonstrated that the entry chamber is not merely a passageway for the substrate. Rather, it is essential for L-aspartate hydroxylation.

### A predicted family of fungal 3-nitropropionate-producing hydroxylases

**Figure 12:**
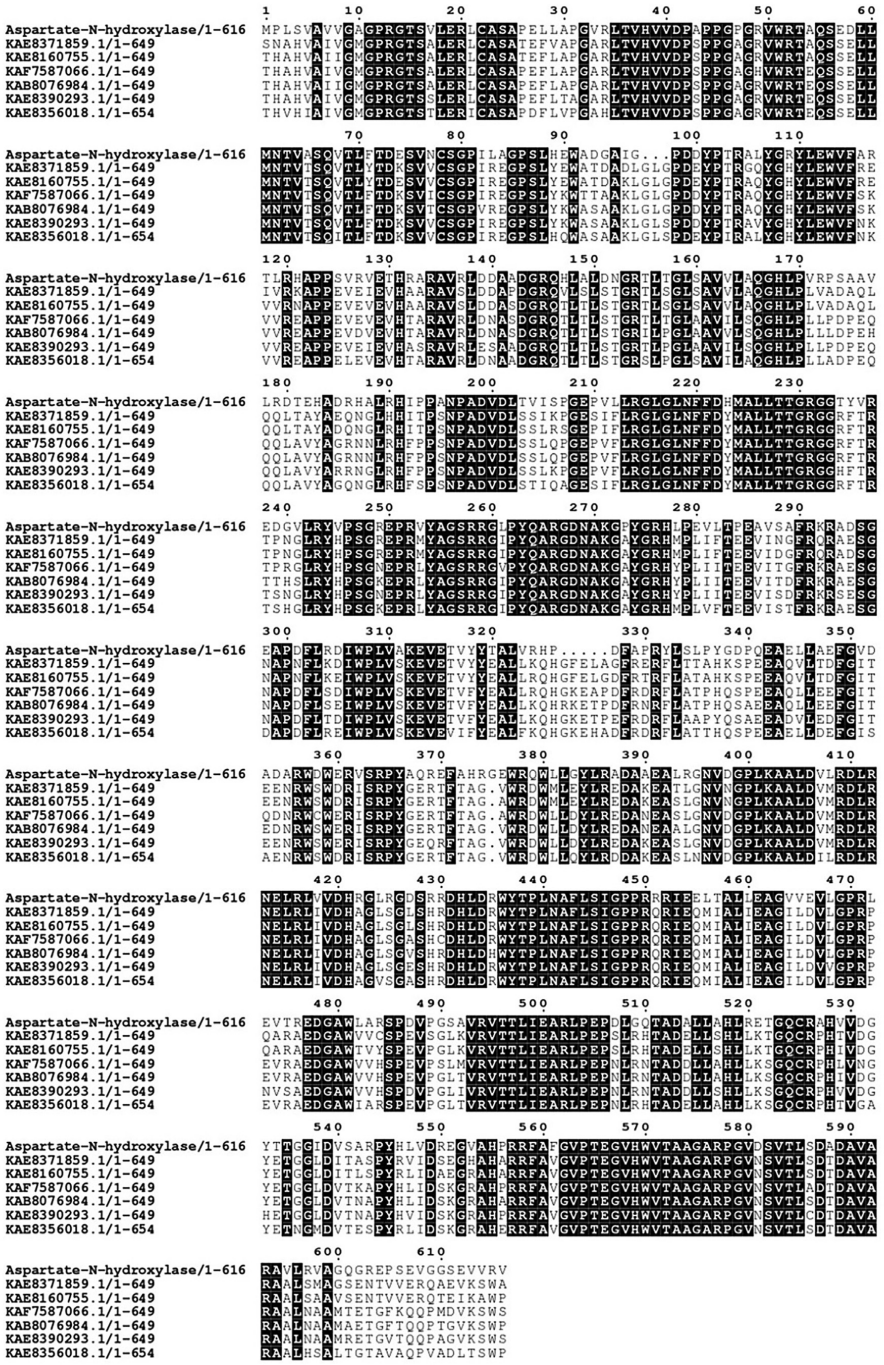
**Alignment of *Streptomyces sp. V2* L-aspartate N-hydroxylase with representative protein sequences retrieved from the genomes of *Aspergillus* species**. All residues forming the aspartate (Fig. 10), NADPH, and FAD (Fig. S4) sites are conserved. KAE8371859 is a hypothetical protein from *Aspergillus bertholletiae*, KAE8160755 from *Aspergillus tamarii*, KAF7587066 from *Aspergillus hancockii*, KAB8076984 from *Aspergillus leporis*, KAE8390293 from *Aspergillus alliaceus*, KAE8356018 from *Aspergillus coremiiformis*.

Many fungi, especially of the *Aspergillus* genus, are well-known producers of 3-nitropropionic acid a potent irreversible inhibitor the mitochondrial succinate dehydrogenase (37, 38, 39). We asked the question of whether this mycotoxin could be produced by fungal homologs of the bacterial L-aspartate hydroxylases. A sequence similarity search confirmed this hypothesis. The genomes of many *Aspergillus* species comprise widely distributed genes encoding for predicted proteins with >70% sequence identities to *Streptomyces* sp. *V2* L-aspartate N-hydroxylase and its homologous bacterial enzymes. All active-site residues forming the FAD, NADPH, catalytic, and entry chamber sites are conserved as can be seen from the alignment shown in Figure 12. Therefore, it can be confidentially predicted that these enzymes are part of the fungal biosynthetic pathway that generates 3-nitropropionic acid (38). This observation expands the role of this family. In bacteria, it is primarily involved in the generation of nitrosuccinate, an HNO_2_ donor for N-N bond biosynthesis, whereas in fungi it is responsible for mycotoxin generation. It is interesting to note the subtle difference between these biochemical roles; in the former case, the product must be protected from decarboxylation, possibly by rapid transfer to the HNO_2_-producing lyase, whilst the decarboxylated compound is the intended final product of the latter process.

### Mechanistic implications

Besides the structural and mechanistic similarity with the class B flavin-dependent monooxygenases (Fig. 1*A*), L-aspartate N-hydroxylase features two characteristic properties: the narrow substrate selectivity and its ability to perform three sequential rounds of hydroxylation with minimal leakage of the intermediate hydroxylated products. The rationale that lies behind the fine substrate recognition can be found in the architecture of the enzyme. L-aspartate N-hydroxylase comprises an additional domain with a helical topology that creates an arginine-rich site for substrate binding (Fig. 8). Remarkably, the crystal structure of the reduced enzyme in complex with the substrate shows that L-aspartate does not firmly bind above the flavin. The substrate rather occupies an entry chamber that is adjacent to the catalytic center but not in direct contact with the flavin. Our mutagenesis experiments show that this site is integral to substrate hydroxylation. This is a notable difference with L-ornithine hydroxylases where the substrate is instead held in front of the flavin with the side-chain amino group in contact with the cofactor C4a atom (16, 17).

These observations lead us to propose that L-aspartate N-hydroxylase and similar enzymes have developed a structural mechanism that allows them to minimize the dissipation of intermediate products and carry on the triple hydroxylation to the final nitro succinate (Fig. 1*B*). The high degree of coupling manifested by the enzyme supports this idea. The entry chamber may represent a sequestration site for the L-aspartate and its incompletely hydroxylated products. A constellation of hydrogen-bonding side chains promotes its tight binding and specific recognition. In the resting enzyme, the “inwards” conformation of Asn62 might hinder further movement of the aspartate into the flavin site. Upon flavin reduction and C4a-hydroperoxyflavin formation, Asn62 might move “outwards”, in the conformation observed in the sulfate complex (Fig. 10). Such a side chain rotation creates the space needed for the peroxyflavin to form and allows the sliding of the substrate into the catalytic core where hydroxylation takes place. The hydroxylated substrate can then be recaptured by the entry chamber where there is space and a proper hydrogen-binding environment for accommodating the oxygenated/hydroxylated intermediate products (Fig. 9*B*). Three of such “release-hydroxylation-recapture” cycles will finally yield nitrosuccinate (Fig. 1*B*). This scheme is attractive because it is compatible with the necessity of repeated events of NADP^+^ release and NADPH binding as needed for triple hydroxylation. They can occur while the hydroxyl-aspartate intermediates are trapped in the entry chamber. Moreover, it also explains how the intermediate and final products are protected from decarboxylation. This is critical for the FzmM and Cre enzymes that are dedicated to the production of the nitrosuccinate directly utilized by the lyase responsible for HNO_2_ generation. Nitrosuccinate can instead non-enzymatically decompose to 3-nitropropionate when the latter is the intended reaction product.

## Experimental procedures

### Recombinant expression

Recombinant (wild-type and Δ14-C-terminal mutant) *Streptomyces* sp. *V2* L-aspartate N-hydroxylase was expressed from pet24a(+) vector by IPTG induction. Transformed BL21(DE3)RP+ cells were grown overnight in LB media supplemented with kanamycin 50 μg/ml. Baffled flasks with 1 L LB media were inoculated with 20 ml of the grown pre-culture and shaken at 37 °C and 200 rpm until an optical density between 0.6 and 0.8 was reached. Each flask was then chilled at 4 °C for 30 min followed by the induction with 1 mM IPTG and moved to 24 °C with 200 rpm shaking for 16 h. The cells were harvested by centrifugation at 5000*g* and stored at −80 °C.

### Purification

Cell pellet from 1 L culture was resuspended in 3× volume of Lysis buffer (Buffer A: 50 mM sodium phosphate pH 8; 300 mM NaCl; 20 mM imidazole; 10% glycerol) supplemented with 1 mM phenyl methyl sulfonyl fluoride (PMSF), 1 mg/ml DNase, leupeptin 10 μm, pepsatine 10 μg/ml and FAD 100 μm. Cells were lysed by sonication, with three cycles (5’’ on and 9’’ off; 1 min each) at 50% amplitude. The insoluble cellular fraction was separated from the soluble lysate by centrifugation at 56,000*g* for 45 min at 4 °C. The cell-free extract was filtered with 0.45 μm filters (Merk) and loaded on 5 ml nickel column, previously equilibrated with lysis buffer, using an Akta System (Cytiva) equipped with a multi-wavelength detector (set at 280/350/450 nm). After loading was completed, the column was washed with buffer A until the absorbance at 280 nm returned to baseline levels. To elute bound proteins a linear gradient of imidazole (20–500 mM) has been applied to the column using elution buffer (buffer B: 50 mM sodium phosphate pH 8; 300 mM NaCl; 500 mM imidazole; 10% v/v glycerol). Fractions containing His_8_-SUMO-aspartate N-hydroxylase were pooled together and concentrated with Amikon 30k up to a suitable volume. The resulting sample was incubated overnight with (1:300) homemade His_6_-SUMO protease (1 mg/ml). While incubating, the protein mix was dialyzed against storage buffer (buffer C: 50 mM Hepes, pH 8, 50 mM NaCl) to remove the excess imidazole. The resulting sample was loaded on a His-Trap column (5 ml, Cytiva) to remove the his-tagged SUMO, while the protein of interest was collected in the flow-through. Prior to crystallization studies, the enzyme was gel filtered with a Superdex 200 10/300 column, previously equilibrated with buffer C. The extinction coefficient of the protein-bound FAD was determined to be 13.80 mM ^−1^ cm^−1^ at pH 7.8.

### Protein crystallization and structure determination

Extensive crystallization screenings were performed with several commercial kits and using an Oryx eight robot (Douglas instruments) in sitting drop plates (SWISSCI, Molecular Dimension). Crystallization droplets were prepared with a 1:1 volume ratio by mixing 15 mg/ml protein in buffer C with the reservoir solution. Initial crystal hits were found with the Δ14-C-terminal mutant in a reservoir solution containing 0.1 M Hepes pH seven and 1.6 M ammonium sulfate. Optimization of the crystallization condition was performed by manually preparing sitting drop plates (Cryschem, Hampton), increasing the drop volume up to 1 μl. Using nylon loops (Hampton Research), crystals were harvested and cryo-cooled in liquid nitrogen. Soaking experiments (0.5 h) with NADPH and L-aspartate were performed in solutions consisting of 9% w/v PEG4000, 1.2 M di-Na tartrate, 28% v/v PEG400, NADPH (1 mM) and L-aspartate (15 mM), followed by flash-freezing in liquid nitrogen. X-ray diffraction data were collected at the European Synchrotron Radiation Facility in Grenoble, France (ESRF). Diffraction images were processed with XDS (40) and Aimless of the CCP4 package (41). Structure solution was performed with Phaser using *in silico*-generated AlphaFold structure as the search model for molecular replacement (31, 42). Atomic models were refined with Refmac5 (43) and Coot (44). Figures were created using ChimeraX (45). Structural superpositions were performed with DALI (35).

### NADPH depletion assay

The activities were monitored following the depletion of NADPH (ε_340nm_= 6.2 mM^−1^ cm^−1^). In a total volume of 150 μl, the reaction mixture contained 1 μM L-aspartate N-hydroxylase, NADPH (0.005–200 μM), and L-aspartate (0.1–15 mM) in 50 mM Hepes, pH 8, 50 mM NaCl. When the NADPH consumption was measured as a function of NADPH concentrations (0.005–200 μM) the concentration of L-aspartate was held constant at 5 mM. When the rate dependence was measured as a function of L-aspartate (0.1–15 mM), the NADPH was kept constant at 100 μM. Data were fit to the Michaelis–Menten equation and analyzed using GraphPad Prism software 9.0. Each point was assayed in triplicate.

### Oxygen consumption assay

The amount of molecular oxygen consumed by L-aspartate N hydroxylase was monitored using a Hansatech Oxygraph system (Norfolk, England). Each assay was performed in 1 ml total volume of 50 mM potassium phosphate pH 7.5 at 25 °C. When the rate of oxygen was measured as a function of NADPH concentrations (0.005–200 μM), the concentration of L-aspartate was held constant at 5 mM. When the rate dependence was measured as a function of L-aspartate (0.1–15 mM), the NADPH was kept constant at 100 μM. The reaction was initiated by the addition of 1 μM of enzyme and monitored for 5 min with constant stirring.

### Pre-steady-state kinetics

The stopped-flow measurements were performed using an SX20 stopped-flow spectrophotometer equipped with a photodiode array detector (Applied Photophysics, Surrey, UK). All solutions were prepared using 50 mM Hepes pH 8 and 50 mM NaCl. Reactions were run in duplicates by mixing 50 μl of each solution at 25 °C. Enzyme solutions were made anaerobic by flushing the vial with nitrogen for 15 min. Moreover, 5 mM of glucose and 0.3 μM of glucose oxidase (*Aspergillus niger*, type VII, Sigma-Aldrich) were added to the solution to remove the leftover oxygen. The enzyme was reduced by mixing 1 to 1.5 equivalent of NADPH. The resulting mixture was then incubated at room temperature until reduction was completed as indicated by the bleaching of the flavin turning transparent. To evaluate the formation of the intermediate, the anaerobically reduced enzyme was mixed with oxygen (130 μM) in the absence and presence of the 1 mM L-aspartate. All data were analyzed using the Pro-Data software (Applied Photophysics) and GraphPad Prism.

### Product characterization

Reaction mixtures (500 μl) were prepared to analyze the reaction products. The sample contained 10 μM L-aspartate N-hydroxylase, 5 mM or 2.5 mM L-aspartate, 5 mM NADPH in 50 mM Hepes pH 8. The reactions were carried out for 2 h at 25 °C with 180 rpm shaking. Control experiments were run with all assay components except the enzyme. To monitor product formation over a time scale of 2 h, aliquots of 50 μl each were taken every 10 min. Subsequently, each sample was diluted with 60 μl 50 mM Hepes pH eight and then denatured with the addition of 100 μl of 100% MeCN HPLC-grade and centrifuged for 10 min at 16,000*g*. This procedure resulted in a 4.2-fold dilution. Samples were analyzed by electrospray ionization quadrupole time-of-flight high-resolution mass spectrometry, ESI-QTOF-HRMS, on an X500B QTOF system (SCIEX, Framingham, MA 01701 USA) equipped with the Twin Sprayer ESI probe coupled to an ExionLC system (SCIEX). The SCIEX OS software 3.0.0 was used as an operating platform. Chromatographic separation was carried out using a bioZen Peptide XB-C18 (150 mm length × 2.1 mm diameter, 2.6 μm particle size; Phenomenex). The mobile phase consisted of water: acetonitrile (1:1) (A) and acetonitrile (B) (both including 0.1% aqueous formic acid, v/v), and the flow rate was 0.3 ml/min. The gradient elution was performed as follows: 2% B at 0.0 to 5.0 min, 2 to 25% B at 5.0 to 11.0 min, 25 to 98% B at 11 to 17 min, 98 to 0.2% B at 17 to 23 min. For MS detection the following parameters were applied: Curtain gas 30 psi, Ion source Gas 1 50 psi, Ion source Gas 2 65 psi, Temperature 600 °C. The full-scan range of m/z 50 to 1000 was monitored in negative mode, with a Spray voltage of −4500 V, a declustering potential of −80 V, and a collision energy of −10 V. Mass calibration was performed with the ESI Negative Calibration solution suitable for the Sciex X500 system (SCIEX), that consists of a mix of known molecular weight chemicals. Qualitative analyses were carried out by overlaying the retention times and mass spectra with standard solutions of L-aspartate and 3-nitropropionate, recorded respectively at 660 and 590 ppm and prepared in HPLC-grade distilled water. The calibration range was 0.15 to 5 mM. Standard solutions of the product were injected in duplicate as described above. Peak assignment was based on retention time and mass spectrum of the reference standard. Quantitation of the analyte was performed by comparing peak areas in a sample chromatogram to the peak areas of the standard dilutions in an external calibration interpolated with the linear regression model.

## Data availability

Coordinates and structure factors have been deposited with accession codes 8CP2 (L-aspartate and NADPH complex) and 8CP5 (sulphate complex).

## Supporting information

This article contains supporting information.

## Conflict of interest

The authors declare that they have no conflicts of interest with the contents of this article.
