# Supplementary material for: A biosynthetic aspartate N-hydroxylase performs successive oxidations by holding intermediates at a site away from the catalytic center
Source: J Biol Chem. 2023 Jun 10;299(7):104904. doi: 10.1016/j.jbc.2023.104904 (PMC10404684; doi:10.1016/j.jbc.2023.104904)
Supplement: Supporting information [file mmc1.pdf]

SUPPLEMENTARY MATERIAL

A biosynthetic aspartate N-hydroxylase performs successive oxidations by holding intermediates at a site away from the catalytic center

Laura Rotilio[a], *Alessandro Boverio*[a,b], *Quoc-Thai Nguyen*[c], *Barbara Mannucci*[d], *Marco Fraaije*[b], *Andrea Mattevi*[a,*]

[a] Department of Biology and Biotechnology, University of Pavia, via Ferrata 9, 27100 Pavia, Italy

[b] Molecular Enzymology, Groningen Biomolecular Sciences and Biotechnology Institute, University of Groningen, The Netherlands

[c] Faculty of Pharmacy, University of Medicine and Pharmacy at Ho Chi Minh City, 41 Dinh Tien Hoang Street, Ben Nghe Ward, District 1, Ho Chi Minh City, Vietnam.

[d] Centro Grandi Strumenti, University of Pavia, Via Agostino Bassi, 21, 27100 Pavia, Italy

[*]**Correspondence to**: Andrea Mattevi, Department of Biology and Biotechnology, University of Pavia, Via Ferrata 1, 27100 Pavia, Italy andrea.mattevi@unipv.it

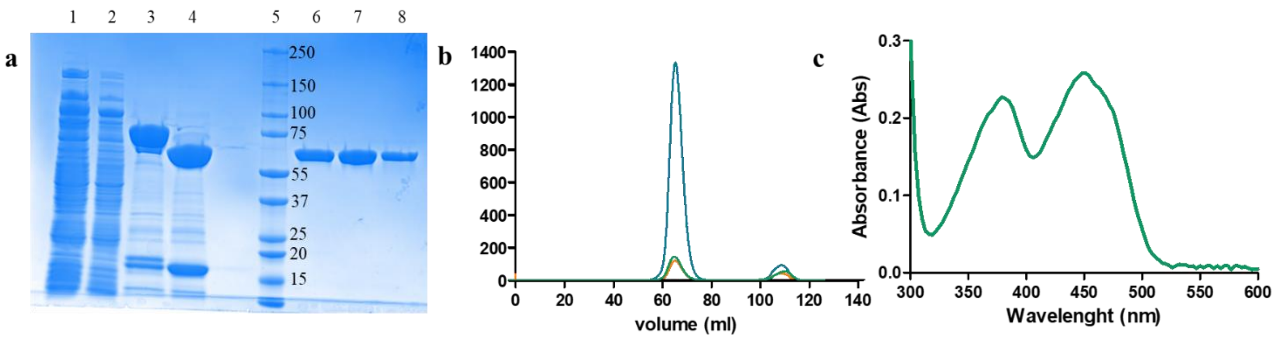

Figure S1. Purification of L-aspartate N-hydroxylase from *Streptomyces sp. V2*. (**A**) SDS-page: lane 1, flow-through; lanes 2, 20 mM imidazole washing; lane 3, IMAC fractions; lane 4, post SUMO-protease cleavage; lane 5, molecular weight markers; lane 6-8, size-exclusion chromatography fractions. (**B**) Size-exclusion chromatography profile of the tag less-protein analysed by a Superdex 200 16/60 column and following the 456 nm absorbance. The elution volume is consistent with a monomeric protein of 65 kDa. (**C**) Uv-Vis absorbance spectrum of the retained oxidized FAD cofactor with absorbance maxima at 370 and 448 nm.

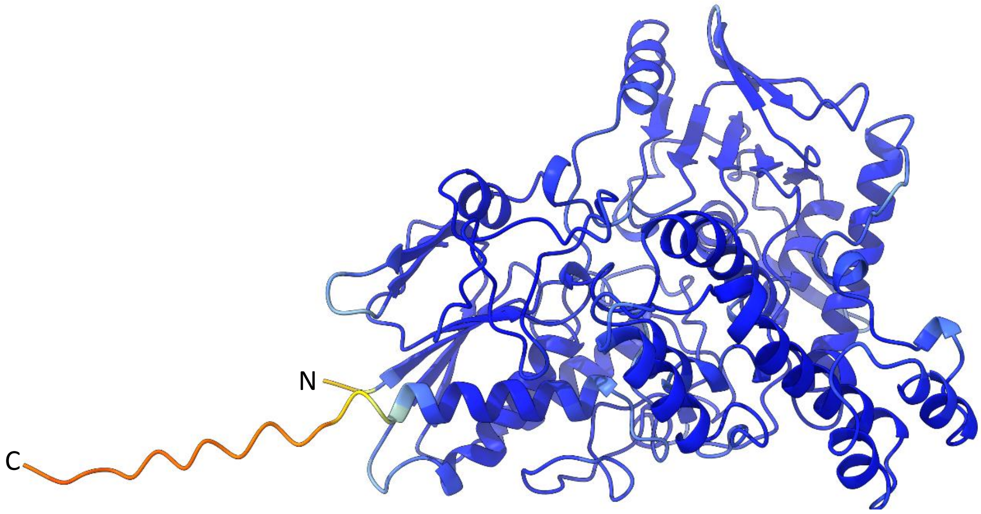

Figure S2. AlphaFold model of *Streptomyces sp. V2* aspartate N-hydroxylase. Residues with a per-residue confidence score below 50 are shown in orange. They are likely unstructured. Based on this model, the C-terminal residues 602-616 were truncated in order to facilitate crystallisation.

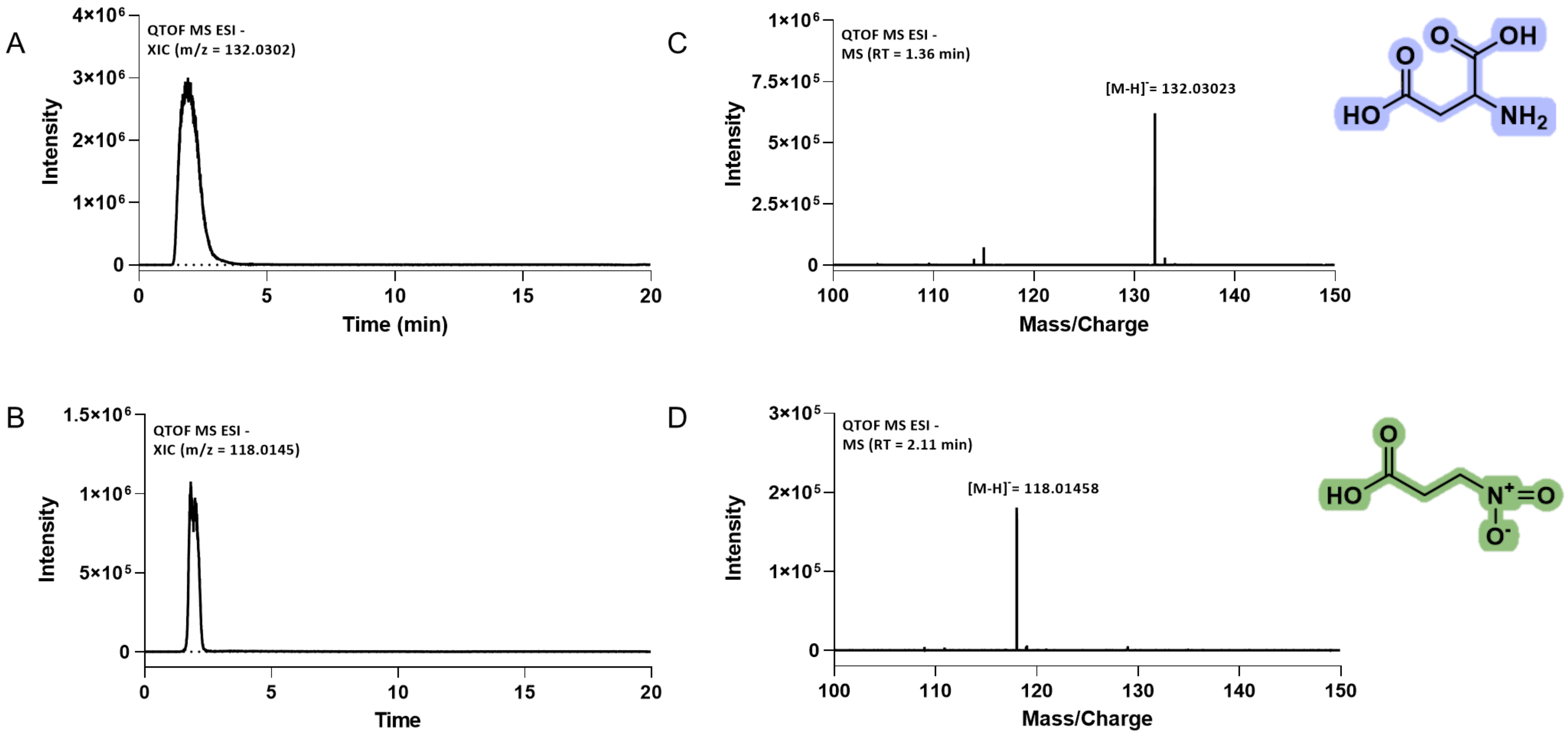

Figure S3. UHPLC-HRMS (negative mode) of substrate and product analytical standards. (A-B) Extracted ion chromatograms (XIC) of the analytical standard at 660 and 590 ppm of L-aspartate and 3-nitropropionate. **(C-D)** ESI-MS spectrum of the $[M-H]^-$ ions (L-aspartate, m/z= 132.03, theoretical m/z = 132.03; 3-nitropropionate m/z= 118.01, theoretical m/z= 118.07).

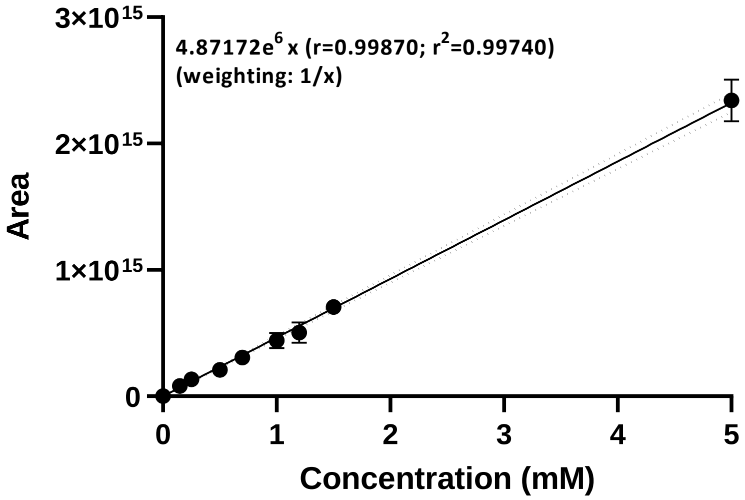

Figure S4. UHPLC-HRMS (negative mode) analyses of 3-nitropropionate analytical standard. The **c**alibration curve in the 0.15 – 5 mM range was interpolated with linear regression.

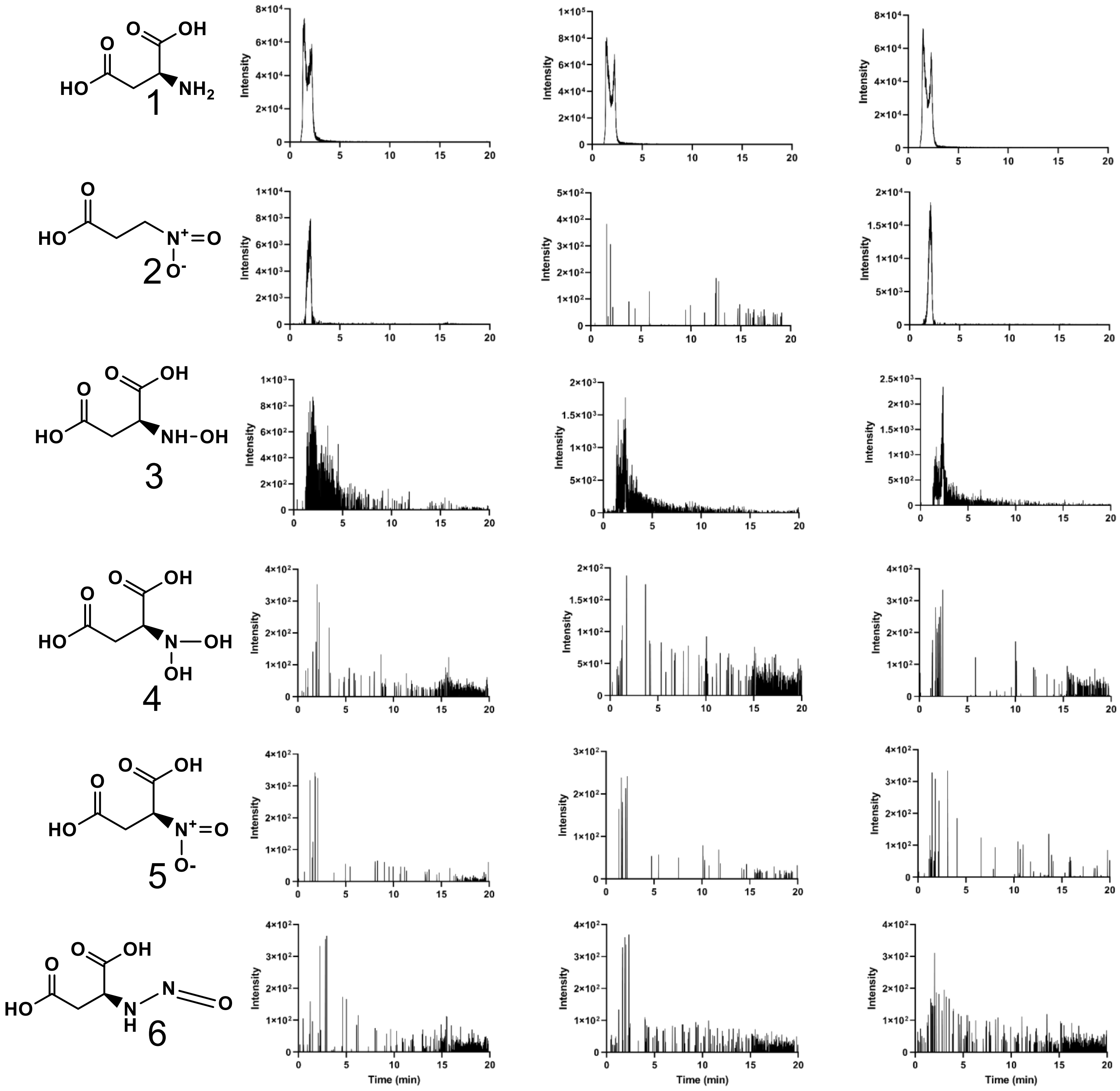

Figure S5. UHPLC-HRMS (negative mode) analyses of *Streptomyces sp. V2* L-aspartate N-hydroxylase reaction mixtures. Extracted Ion (XIC) chromatograms of the reaction mixtures for the wild-type (left panel), N413E (middle panel), and V571F (right panel) proteins. A peak corresponding to L-aspartate (**1**) is present in all reaction mixtures whereas 3-nitropropionic acid (**2**) forms in the experiments with the wild-type and V571F enzymes. Hydroxy-L-aspartic acid (**3**), N,N-dihydroxy-L-aspartic acid (**4**), nitrosuccinate (**5**), and nitroso-L-aspartic acid (**6**) were not detected in any experiment.

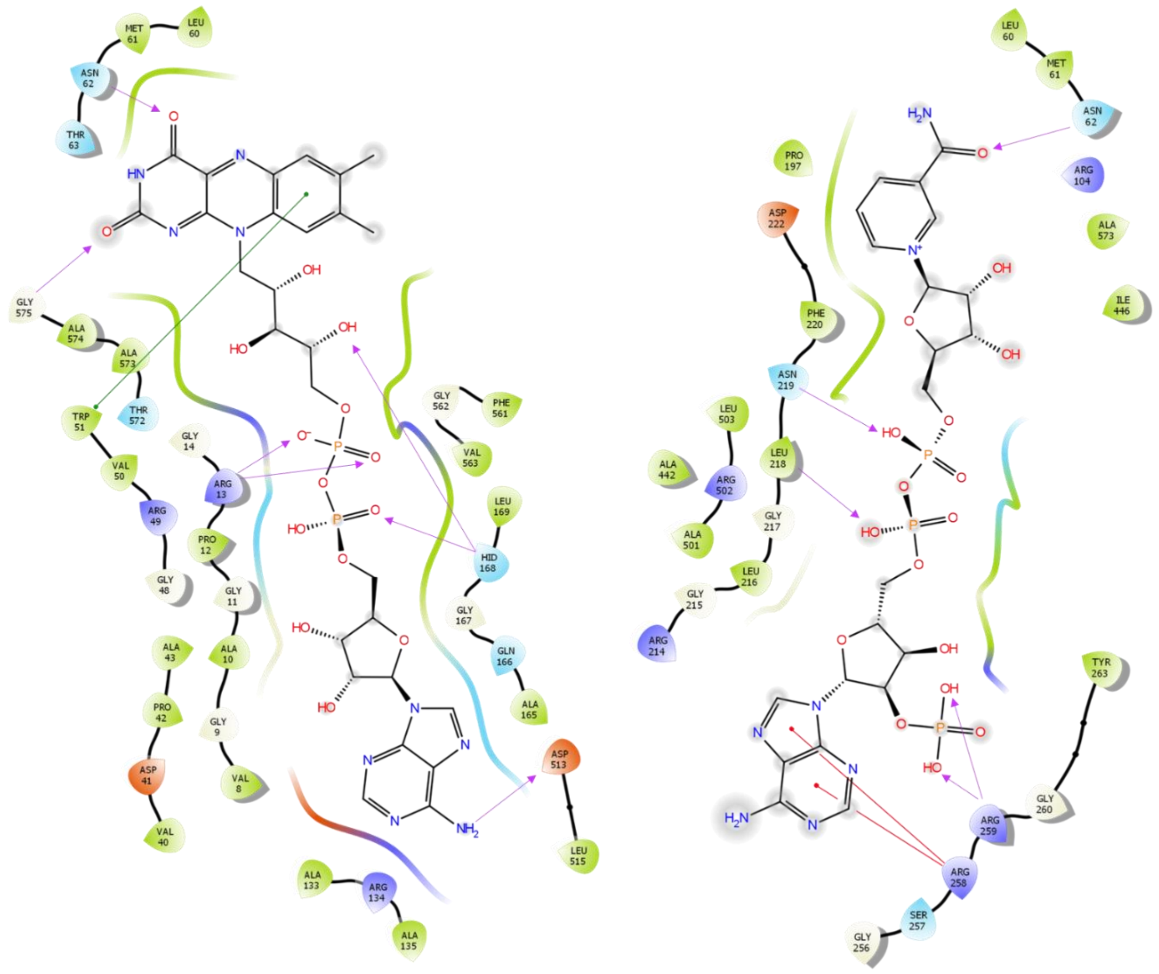

Figure S6. Two-dimensional interactions diagram of the interactions established by the FAD (left) and NADP$^+$ (right) with the protein residues.
